# Nuclear morphometry and chromatin textural characteristics of basal cell carcinoma*

**DOI:** 10.1590/abd1806-4841.20154076

**Published:** 2015

**Authors:** Paola Jung Mendaçolli, Gabrielli Brianezi, Juliano Vilaverde Schmitt, Mariângela Esther Alencar Marques, Hélio Amante Miot

**Affiliations:** 1 Universidade Estadual Paulista "Júlio de Mesquita Filho" (Unesp) - Botucatu, SP, Brazil

**Keywords:** Carcinoma, basal cell, Chromatin, Karyometry, Image processing, computer-assisted, Prognosis

## Abstract

Histological subtypes of basal cell carcinoma have biological, evolutionary and distinct prognostic behavior. The analysis of characteristics of the nucleus can provide data on their cellular physiology and behavior. The authors of this study evaluated nuclear morphological parameters and textural patterns of chromatin from different subtypes of basal cell carcinoma: nodular (n=37), superficial (n=28) and sclerodermiform (n=28). The parameters were compared between neoplasms' subtypes and with unaffected adjacent basal epithelium. Nuclear area and diameter of sclerodermiform neoplasms were superior to the other subtypes. Chromatin's color intensity and fractal dimension were less intense in superficial subtypes. Nuclear roundness and chromatin's entropy presented lower values in tumors than in normal epithelium. There was significant correlation between morphological and textural variables of normal skin and tumors. Morphometric elements and textural chromatin's homogeneity of basal cell carcinomas may be related to evolutionary, biological and behavior particularities related to each histotype.

## INTRODUCTION

Basal cell carcinoma (BCC) is the most common malignancy among men, and its incidence is on the rise in several countries.^1-3^ At Faculdade de Medicina de Botucatu-SP (Brazil) more than 900 patients are operated a year.

There are few reported cases of BCCs metastasis, which leads to a survival rate close to 100%, as long as it is adequately treated. However, it has high local malignancy, growing slowly and progressively, and may expand into surface and/or invade underlying tissues such as muscle, cartilage and bones, causing destruction and deformation of the area, implying in high morbidity.^1^

Its histogenesis is not completely unveiled. It consists of cells similar to basaloid cells of the epidermis, however, there are elements indicating that it originates from immature pluripotent epithelial cells, unable to differentiate them and to keratinize normally.^1,4^

BCCs have different clinical and histopathological subtypes that present distinct evolutionary biological behavior and prognosis. The main histological subtypes are nodular (which can also present with cystic and pigmented component), sclerodermiform, superficial, infiltrative and rare variants: micronodular and fibroepithelioma of Pinkus.^5,6^

Evolutive differences of the various types of BCC are not well defined. The 3 subtypes that best represent the tumor are nodular, sclerodermiform and superficial. Despite more than 30% of lesions present mixed components, different kinetic characteristics, invasiveness and relapses are observed, which refer to subtypes, suggesting different biological behavior.

Sclerodermiform, infiltrative and micronodular forms are considered of infiltrative growth, with more aggressive clinical behavior and increased risk of relapse. Superficial, pigmented, and cystic forms are considered of expansive growth, with milder behavior. Superficial BCCs with multifocal characteristics tend to recur if the margin of excision is meager, and if there are neoplastic nests interspersed with health epithelium areas.^1,7^

Morphological analysis of cell nuclei by histology can provide data on the physiology of the cell and contribute to the study of the diagnosis and prognosis of neoplastic lesions. Similarly, changes in the cell cycle or metabolism due to pharmacological, physiological or epigenetic action are accompanied by alterations in the architecture of the nuclear chromatin. Thus, nuclear morphology and texture characteristics have been studied as prognostic factors in many neoplasms.^8-11^ However, to date there is no research on nuclear morphometry and chromatin heterogeneity of different subtypes of BCCs.

In this study, the authors aimed to evaluate nuclear morphometric characteristics (area, perimeter, circularity and larger diameter), intensity and heterogeneity of chromatin texture (fractal dimension, image entropy and texture-Ra estimator) between different histological subtypes of BCC, and between cancer and basal keratinocytes from normal adjacent epithelium. Also, they explored the correlation between the morphometric variables and chromatin texture.

## METHODS

Cross-sectional study involving 120 BCCs selected from registered patients in the pathology department of Faculdade de Medicina da Unesp de Botucatu, from 2010 to 2012, based on histopathology reports. Initially, we had 40 nodular BCC, 40 surface BCC and 40 sclerodermiform BCC. Among them, we chose only the ones with most characteristic morphology of each subtype and better image quality, restricting the study to 37, 28 and 28 tumors in each group (N = 93). The Ethics Committee of the institution approved the study.

**Figure 1:**
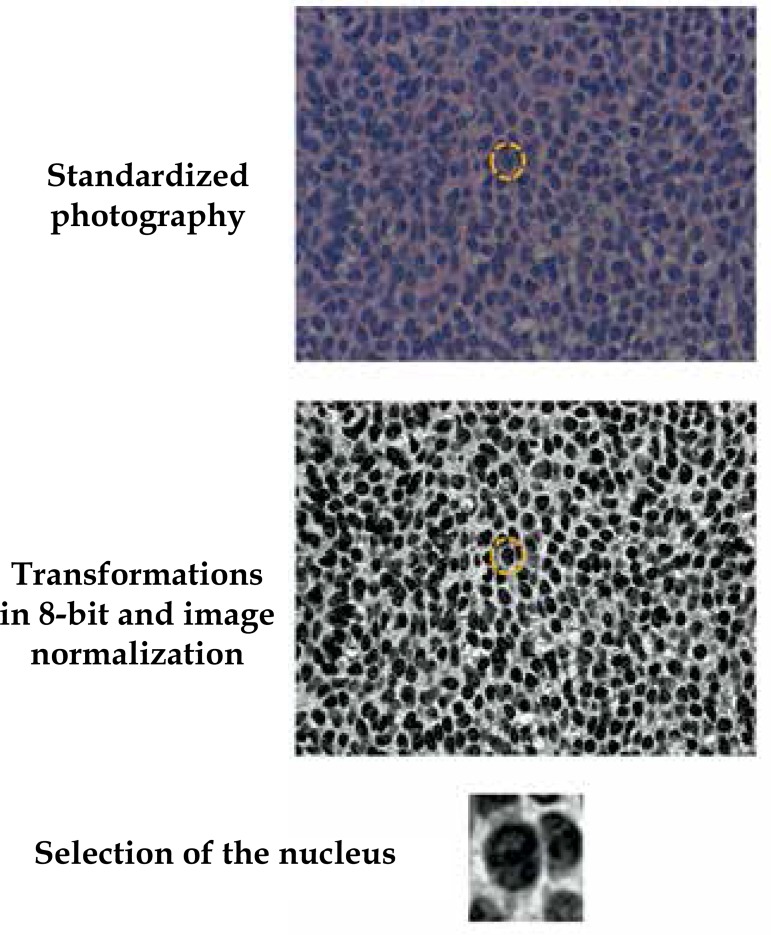
Representation of the acquisition and pre-processing of nuclei

Slides were photographed in order to record 30 well-individualized nuclei of each BCC and 30 nuclei in the basal layer of the normal adjacent epithelium. After that, these nuclei were cut and the resulting images were transformed to 8-bit (256 grayscale), standardized and subjected to analysis of entropy, fractal dimension (cube box-count technique) and Ra (Figure 1).^12^ Morphological aspects of the nucleus, such as circularity, larger diameter, perimeter and area were also assessed. Analyses were performed by ImageJ 1.46 software and its specific plugins for each index.^13^

Variables were tested for normality by Shapiro-Wilk test. Variables related to tumors groups were represented by mean and standard deviations or medians and quartiles, if indicated, and compared using generalized linear mixed-effects model.^14^ Correlations between variables were estimated by Spearman coefficient if the distributions were not parametric.^14^

Data were tabulated in MSExcel and analyzed by SPSS 20.0 software.^15^ A two-tailed p value <0.01 was considered significant.

## RESULTS

We evaluated 5580 nuclei from 93 tumors and their adjacent epithelium. The main variables evaluated are arranged in table 1.

**Table 1:** Main variables evaluated in the study (mean and standard deviation) according to the cancer subtype and adjacent epithelium

	Nodular (N=37)	Sclerodermiform (N=28)	Superficial (N=28)	Epithelium (N=93)
Area*	65.4 (23.1)	73.9 (29.6)	66.6 (21.8)	64.6 (17.6)
Perimeter**	32.9 (7.7)	34.3 (7.9)	32.7 (6.1)	33.6 (5.9)
Circularity	0.77 (0.12)	0.78 (0.12)	0.78 (0.11)	0.73 (0.11)
Larger diameter**	11.7 (2.7)	12.5 (2.9)	11.9 (2.5)	12.5 (2.2)
Intensity	62.7 (24.4)	61.5 (25.9)	54.8 (20.9)	43.5 (16.1)
Ra	236034.4 (7663.2)	233579.6 (12220.8)	234991.8 (8207.5)	234794.4 (5990.7)
Entropy	5.6 (0.51)	5.7 (0.58)	5.6 (0.55)	5.3 (0.53)
Fractal dimension	2.48 (0.05)	2.50 (0.05)	2.45 (0.05)	2.45 (0.05)

* micra2;

** micra

Correlations between the evaluated nuclear variables within the epithelium and within tumors are displayed in tables 2 and 3. We highlight the significant correlation between kariometric and textural rates both in neoplastic epithelium and in adjacent epidermis.

**Table 2:** Correlation (Spearman's rho) between the nuclear variables evaluated in normal epithelium

	Perimeter	Circularity	Larger diameter	Intensity	Ra	Entropy	Fractal
Area	0.87*	0.12	0.76*	0.43*	0.90*	0.23	0.70*
Perimeter	-	0.55*	0.90*	0.47*	0.73*	0.03	0.03
Circularity	-	-	0.56*	0.24	0.02	0.045*	0.16
Larger diameter	-	-	-	0.39	0.65*	0.12	0.47*
Intensity	-	-	-	-	0.22	0.23	0.60*
Ra	-	-	-	-	-	0.17	0.52*
Entropy	-	-	-	-	-	-	0.10

* p<0.01

**Table 3:** Correlation (Spearman's rho) between the nuclear variables assessed in the tumors

	Perimeter	Circularity	Larger diameter	Intensity	Ra	Entropy	Fractal
Area	0.92*	0.10	0.84*	0.40*	0.89*	0.24	0.70*
Perimeter	-	0.45*	0.92*	0.40*	0.79*	0.02	0.65*
Circularity	-	-	0.45*	0.05	0.05	0.56*	0.09
Larger diameter	-	-	-	0.28	0.74*	0.08	0.48*
Intensity	-	-	-	-	0.26	0.32	0.67*
Ra	-	-	-	-	-	0.21	0.56*
Entropy	-	-	-	-	-	-	0.24

* p<0.01

When we compared tumor epithelia with basal keratinocytes and different subtypes, few variables demonstrated differences between them (Table 4), especially larger area and nuclear diameter of sclerodermiform, smaller intensities of chromatin, and fractal dimension between superficial. In addition, tumors presented higher entropy of chromatin and nuclear circularity.

**Table 4:** F coefficient (p value) of the regression of different variables comparison (generalized linear mixed-effects model)

	Tumor x Normal	Type of tumor	Observation
Area	3,30 (0,07)	5,33 (0,01)	Sclerodermiform higher than others
Perimeter	0,35 (0,55)	4,21 (0,02)	
Circularity	41,23 (0,00)	3,57 (0,03)	Lower circularity in normal epithelium
Larger diameter	6,73 (0,01)	6,43 (0,00)	Sclerodermiform higher than others
Intensity	94,90 (0,00)	4,90 (0,01)	Superficial lower than others
Ra	0,49 (0,48)	3,44 (0,03)	
Entropy	73,24 (0,00)	0,71 (0,49)	Lower entropy in normal epithelium
Fractal dimension	36,12 (0,00)	6,91 (0,01)	Superficial lower than others

## DISCUSSION

There are nuclear morphometric elements and chromatin texture that differentiate BCC from adjacent basal epithelium and from its subtypes. Changes of nuclear morphology and chromatin texture are classically described by pathologists as criteria for tissue differentiation and tumors.^8,16^

Basal epidermal keratinocytes were less circular and presented lower entropy, added to the fact that the standard deviation of their measurements is lower than the same index of BCCs. All these factors point towards organization and homogeneity of normal epithelium compared with neoplastic.

Usually, nuclear alterations are observed in the characterization of neoplastic tissue (e.g.: change of shape, intensity, nucleolus and chromatin distribution), and nuclear aberrations are associated with malignancy and tumors survival. However, tissues in normal development or under epigenetic control (e.g., ultraviolet radiation, exposure to hormones) may also have measurable nuclear alterations, representing the intensity of metabolic activity.^8,12,17^

In this study, there was a significant correlation between various morphological variables, suggesting that the nucleus phenotypic changes are not specific to one or another morphometric marker, but occur simultaneously for characterizing a phenomenon. Multivariate models that simultaneously explore different nuclear variables may have higher discriminating behavior than the independent analysis of each variable.

In the cases studied, significant differences between normal epithelium and neoplastic tissue were identified as circularity and entropy. This suggests that there is differentiation of nuclear morphology and chromatin distribution in tumor tissue as a whole, relative to epidermis. Moreover, even among tumors subtypes we found differences in the nuclear area, larger diameter, intensity and fractal dimension of chromatin, which may represent genomic differences or metabolic activity that justify independent biological behavior among histological subtypes.^8,10,17^

Superficial forms, which have a lower degree of invasiveness, showed smaller fractal dimension and chromatin intensity. This can be reflected by the type of growth, more prominently horizontal and in a slurred way, of these histotypes.

Sclerodermiform type, with evident infiltrative behavior, showed higher morphometric rates of nucleus size. In contrast to the superficial forms, early and insidious invasiveness of the dermis can be represented by a greater biological activity of these histotypes.

This is a preliminary study suggesting morphometric differences in chromatin textural characteristics of BCCs and adjacent skin. Later, these rates should be estimated to study prognostic aspects of BCC, even within the same histotype.^8,10,18^ Authors have not had a sufficient number of BCC recurrence cases with analytical quality for this project, but this should be further investigated.

Other staining methods with stoichiometric characteristics with DNA, as silver or Feulgen, may contribute to the exploitation of kariometric variables and chromatin texture with prognostic characteristics for this study.^18-20^

## CONCLUSION

There were characterized aspects of nuclear morphometry and textural characteristics of BCC chromatin, and identified elements that differentiate BCC from adjacent epithelia and from their subtypes, which may be related to their evolutionary biological behavior characteristics.
